# Modeling Vestibular Compensation: Neural Plasticity Upon Thalamic Lesion

**DOI:** 10.3389/fneur.2020.00441

**Published:** 2020-05-22

**Authors:** Stefan Reuss, Elena Siebrecht, Ulla Stier, Hans-Georg Buchholz, Nicole Bausbacher, Nadine Schabbach, Andrea Kronfeld, Marianne Dieterich, Mathias Schreckenberger

**Affiliations:** ^1^Department of Nuclear Medicine, University Medical Center, Johannes Gutenberg-University, Mainz, Germany; ^2^Department of Anatomy and Cell Biology, University Medical Center, Johannes Gutenberg-University, Mainz, Germany; ^3^Department of Neuroradiology, University Medical Center, Johannes Gutenberg-University, Mainz, Germany; ^4^Department of Neurology and German Center for Vertigo and Balance Disorders, Ludwig Maximilians-University München, Munich, Germany; ^5^Cluster of Systems Neurology, SyNergy, München, Germany

**Keywords:** PET-imaging, lesion, cerebral cortex, thalamus, neuronal tracing, immunofluorescence

## Abstract

The present study in rats was conducted to identify brain regions affected by the interruption of vestibular transmission and to explore selected aspects of their functional connections. We analyzed, by positron emission tomography (PET), the regional cerebral glucose metabolism (rCGM) of cortical, and subcortical cerebral regions processing vestibular signals after an experimental lesion of the left laterodorsal thalamic nucleus, a relay station for vestibular input en route to the cortical circuitry. PET scans upon galvanic vestibular stimulation (GVS) were conducted in each animal prior to lesion and at post-lesion days (PLD) 1, 3, 7, and 20, and voxel-wise statistical analysis of rCGM at each PLD compared to pre-lesion status were performed. After lesion, augmented metabolic activation by GVS was detected in cerebellum, mainly contralateral, and in contralateral subcortical structures such as superior colliculus, while diminished activation was observed in ipsilateral visual, entorhinal, and somatosensory cortices, indicating compensatory processes in the non-affected sensory systems of the unlesioned side. The changes in rCGM observed after lesion resembled alterations observed in patients suffering from unilateral thalamic infarction and may be interpreted as brain plasticity mechanisms associated with vestibular compensation and substitution. The second set of experiments aimed at the connections between cortical and subcortical vestibular regions and their neurotransmitter systems. Neuronal tracers were injected in regions processing vestibular and somatosensory information. Injections into the anterior cingulate cortex (ACC) or the primary somatosensory cortex (S1) retrogradely labeled neuronal somata in ventral posteromedial (VPM), posterolateral (VPL), ventrolateral (VL), posterior (Po), and laterodorsal nucleus, dorsomedial part (LDDM), locus coeruleus, and contralateral S1 area. Injections into the parafascicular nucleus (PaF), VPM/VPL, or LDDM anterogradely labeled terminal fields in S1, ACC, insular cortex, hippocampal CA1 region, and amygdala. Immunohistochemistry showed tracer-labeled terminal fields contacting cortical neurons expressing the μ-opioid receptor. Antibodies to tyrosine hydroxylase, serotonin, substance P, or neuronal nitric oxide-synthase did not label any of the traced structures. These findings provide evidence for opioidergic transmission in thalamo-cortical transduction.

## Introduction

The bilateral central vestibular system provides an excellent paradigm to study mechanisms of brain plasticity, since it is able to compensate a certain loss of function by reorganizing its neurotransmission parameters (“vestibular compensation,” VC). Patients suffering from an acute deficit of vestibular function caused by unilateral peripheral or central lesions show symptoms such as vertigo, dizziness, nystagmus, or body and perceptual tilts that may, however, be relieved or disappear completely in the course of the lesion. This is thought to be due to the compensation of unilateral functional loss by changes in neurotransmission such as up- or down-regulation of transmitter and/or receptor synthesis or affinity, to the shift of transmission to other brain regions in the same or other hemisphere and to reactive neuro- and gliogenesis [cf. (1–3)].

Our knowledge of vestibular processing in cortical and subcortical cerebral regions, however, is restricted. Some studies describe functional aspects of these regions during VC, based on neuroanatomical, physiological, and imaging studies in animal models and on patient studies investigating changes in cerebral metabolism after vestibular lesions (4–14).

In animals, neuronal plasticity underlying VC include changes in the physiological properties of afferents in the end-organ, mediated through the efferent system, as well as changes in the function of the vestibulocerebellum and its projection to the brainstem vestibular nuclei [VN; (15–17)]. These involve functional changes in neurotransmitter systems using ɤ-amino-butyric acid (GABA), acetylcholine (ACh), glutamate and, in particular, dopamine, and their receptors [cf. (18–20)]. These effects were observed in the VN, the target structure of the vestibular organ, in studies focusing on intact central processing structures and compensation following experimental labyrinthectomy in animal models (6, 14, 18–25) or in acquired peripheral lesions in patients (11, 13, 14, 26, 27).

It is, however, open so far as to which aspects of VC occur when the end-organ is left intact and instead the VN-cortical stream is experimentally interrupted (corresponding to a thalamic stroke in patients). There is evidence that “multiple, parallel plastic processes at various sites in the brain” including cerebellum, entorhinal cortex, and hippocampus, are involved in vestibular compensation of central lesions [cf. (14)].

Only a few studies have utilized an *in vivo*-approach to vestibular processing in animals, and most of these were electrophysiological studies. Less is known about processing of vestibular input after lesion of subcortical vestibular regions in animals, and functional brain imaging such as PET was so far used only in clinical studies. A distinct pattern of activation and inactivation of cerebro-cortical and subcortical regions has been observed in patients suffering from vestibular neuritis (10, 12), unilateral medullary and midbrain strokes (28, 29) or from unilateral infarction of the posterolateral thalamus (a relay-station for vestibular input to the vestibular cortex). The latter caused a significant reduction of the activity within the multisensory vestibular cortex areas in the hemisphere ipsilateral, and to a lesser extent also contralateral, to the thalamic lesion (11).

Our previous study was the first to use functional brain imaging (positron emission tomography, PET) upon galvanic vestibular stimulation (GVS) in rats to identify brain regions involved in vestibular processing (30). Cortical and subcortical clusters of augmented rCGM were observed in primary and secondary somatosensory cortex, auditory cortical areas, cingulate gyrus, dorsal intermediate entorhinal cortex, granular insular cortex (to minor extent), hippocampus, and amygdala in the left hemisphere upon either left or right vestibular stimulation. Bilateral activation was found in the cerebellar crus 1 and within the laterodorsal thalamic nucleus in both its dorsomedial and ventrolateral parts (LDDM, LDVL). Our data revealed that these thalamic structures possess major functions such as to collect, filter, integrate, and send vestibular information to cortical regions and combine it with other sensory modalities. To affect the major relay station of the thalamo-cortical vestibular network, these thalamic subnuclei were lesioned unilaterally in the present study.

The availability of pre-lesion testing and well-defined lesions that may later be characterized by histology are major benefits of functional brain imaging in experimental animals. Thalamic lesions, as conducted here, have the advantage over peripheral lesions that they do not affect the peripheral auditory system in a way labyrinthectomy does. They reduce respective artifacts and, in particular, they mimic the situation of thalamic infarction in patients.

The aim of the present study therefore was in a first part to investigate the processing and compensatory mechanisms at the cerebro-cortical and subcortical level after a defined central vestibular lesion and during the recovery phase in rats.

A second series of experiments was conducted to further investigate the connections between subcortical and cortical regions known from imaging studies to process vestibular information. We selected the somatosensory (S1) region and the anterior cingulate cortex (ACC) for injection of a *retrograde* neuronal tracer to label afferent subcortical neurons. We then injected *anterograde* tracer substances into thalamic nuclei (parafascicular, ventral posterolateral/-medial, laterodorsal) to label cortical target sites. Selected sections exhibiting anterograde or retrograde labeling were processed for immunohistochemistry to test for the possible presence of tyrosine-hydroxylase, serotonin, neuronal nitric oxide-synthase, substance *P*, or the μ-opioidergic receptor in or adjacent to tracer-labeled structures.

## Materials and Methods

The procedures concerning animals reported in this study complied with German and European laws for the protection of animals and were approved by the county-government office (Bezirksregierung Rheinhessen-Pfalz). All efforts were made to minimize the number of animals and their suffering.

### Anesthesia

Galvanic stimulation and PET-scans, electrolytic thalamic lesions, magnetic resonance imaging, and intracerebral injections of neuronal tracers in rats were conducted at day time under general anesthesia. This was initiated by isoflurane inhalation and then maintained by 5 mg/kg b.wt. of a mixture of 55% ketamine (Ketavet, Pfizer, Berlin, Germany) and 45% xylazine (Rompun, Bayer, Leverkusen, Germany) given intraperitoneal (i.p.). After 25 min, rats received 30% of the initial dose to continue anesthesia. Animals were placed under a red light to keep body temperature constant.

### First Experimental Series: PET Study

#### Animals

Nineteen adult male Sprague-Dawley rats (Harlan-Winkelmann, Borchen, Germany) were maintained under constant conditions (light:dark 12:12 h, room temperature 21 ± 1°C) with food and water available *ad-libitum*. Body weights were 286 ± 57 g (mean ± SD, range 230–400 g) at first experimental day, and 429 ± 37 g (range 340–470) at the final experimental day.

### Galvanic Vestibular Stimulation

Galvanic vestibular stimulation (GVS) was used to stimulate the vestibular system. This method, a variant of transcranial direct current stimulation, is well-established for vestibular stimulation in animal models (31) and for vestibular diagnosis and therapy in men (32–34).

As described in detail in our previous paper (30), the stimulation (cathodal) electrode was placed subcutaneously in the midline, and the anodal electrode was placed at the left external auditory meatal cartilage. The electric stimulus, generated by a custom-built battery-driven stimulator, consisted of square-wave pulses (1 Hz; 0.2 mA, 500 ms duration, 3,000 stimuli). The effectiveness of GVS was evaluated by the presence or absence of a resulting nystagmus in the animals. Immediately before the 50 min stimulation period, the tracer ^18^F-FDG was injected.

### Thalamic Lesions

The animals were divided into two groups. One group consisted of eight rats receiving a thalamic lesion, i.e., damage of the left dorsomedial (LDDM) and ventrolateral (LDVL) parts of the laterodorsal thalamic nucleus. The stereotactic coordinates for LDDM/LDVL lesion (centered at Bregma−3 mm, 2 mm lateral to midline, 4.5 mm below surface) were taken from the rat brain atlas (35). The animal's head was fixed in a prone position in a stereotaxic unit. A little hole was drilled into the skull using a dental drill. The electrode was inserted stereotactically and a current of 1.5 mA lasting 10 s was applied. After replacing the electrode, the animals received adequate medical treatment. Four sham-lesioned (SL) control animals underwent the same procedure without applying a current.

Immediately after surgical intervention, (sham-) lesion sites were verified by magnetic resonance imaging (MRI) using a Siemens Magnetom 3.0 Tesla (Siemens, Erlangen, Germany). For this purpose, a 3D-gradient echo data set with TR = 50 ms, TE = 43 ms, FA = 25° and an isotropic resolution of 0.3 mm was acquired. The puncture was observed as a hypo-intense, elongated volume, and was marked in a copy of each 3D-data set. After normalization of each original data set, transformations were transferred to the copies, which were then averaged.

After the final PET-scan, animals were killed by cervical dislocation. The brains were removed and deep-frozen, cut on a cryostat in the frontal plane and stained with hematoxylin-eosin for lesion verification.

### PET-Scans

The data were acquired with the small-animal PET-scanner micro-PET Focus 120 (Siemens, Erlangen, Germany). The scanning procedure was the same as described in our previous study (30). Animals were deprived of food in the evening prior to scanning. Listmode acquisition started at 60 min after tracer injection and data were collected for 30 min. A transmission scan was performed using a ^57^cobalt source to correct for radiation attenuation of tissues of different densities. A 3D maximum a posteriori (MAP) algorithm with 18 iterations and a regularization parameter of 0.05 within a 256 × 256 matrix was used to reconstruct images with scatter and attenuation correction. The PET-scanner consisted of 168 lutetium-oxyorthosilicate detectors ordered in four contiguous rings of 25.8 cm in diameter and 7.6 cm axial length resulting in an intrinsic spatial resolution of 1.4 mm at central field of view (CFOV) and a slice thickness of 0.8 mm (36).

**Figure 1:**
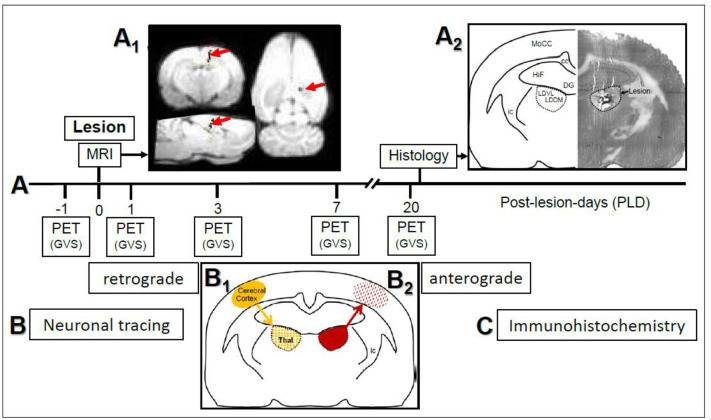
**(A)** Study design and time course of μPET-scans in rats upon galvanic vestibular stimulation (GVS). An electrolytic lesion was conducted in the thalamus (dorsomedial and ventrolateral parts of laterodorsal thalamic nucleus, LDDM, LDVL), followed by magnetic resonance imaging (MRI) scan to control for lesion site (red arrows in **A**_**1**_). Control animals received sham-lesions. PET-scans were conducted 1 day before the lesion and on post-lesion days 1, 3, 7, and 20. After the final scan, brains were processed for lesion histology. The approximate level exhibiting the lesion site is Bregma−3 mm, according to Figure 58 of the rat brain atlas (35). All procedures were carried out under deep anesthesia. Details of the GVS paradigm were given in Best et al. (30). **(B)** Neuronal tracing; retrograde labeling of thalamic neurons upon cortical injections **(B**_**1**_**)** and anterograde labeling of cortical afferents upon thalamic injections **(B**_**2**_**)**. **(C)** Selected sections were exposed to immunohistochemical incubations for transmitter and receptor molecules. cc, corpus callosum; DG, dentate gyrus; HiF, hippocampal formation; ic, internal capsule; MoCC, motor regions of cerebral cortex.

To examine the chronic course of metabolism after lesion, each animal was scanned upon vestibular stimulation 1 day before the thalamic lesion and four times after lesion (i.e., at post-lesion-days one, three, seven, and twenty, see Figure 1). Brain activity was evaluated using the radiopharmaceutical tracer ^18^Fluorodesoxyglucose (^18^F-FDG), a glucose-analog containing the positron-emitting radioactive isotope fluorine 18. Animals received i.p. injections of 25–35 MBq FDG prior to each stimulation. The tracer was taken up and accumulated in brain regions relative to their metabolic rates.

### PET-Data Analysis

From the 19 rats used in this experimental series, seven were excluded from analysis since they were used for lesion testing prior to the experiments or were not scanned at all time-points for technical reasons. Data from eight lesioned and four sham-lesioned animals were analyzed as follows.

Spatial normalization into the in-house FDG-template in Paxinos space and statistical analyses of rat brain images were performed with a tailored version of SPM5 (Wellcome Department of Cognitive Neurology, London, UK, http://www.fil.ion.ucl.ac.uk/spm), as described by Best et al. (30). A three-dimensional Gaussian filter using 8 mm full width at half-maximum kernel (voxel size, 2 × 2 × 2 mm^3^) was used for smoothening.

Two different comparisons were analyzed: first, paired *T*-tests between each PLD (1, 3, 7, or 20 days after surgery) and the pre-lesion status and the two-samples *T*-tests between the lesion and the sham-group at each PLD. Statistical thresholds were initially set to *P* < 0.001 (uncorrected). Multiple comparison correction of the clusters at *P* < 0.001 was performed using the small volume correction (SVC) method implemented in SPM. Clusters were considered as significant if *P* (SVC-corrected) < 0.05.

For illustrating purposes, the activations and deactivations, respectively, were superimposed on standard MRI-templates in Paxinos-space.

### Second Experimental Series: Neuroanatomical Study

#### Animals

Twenty-five rats (five groups of five animals each) were used in this part of the study. Each animal of a given group received a single application of one of the neuronal tracer substances into one of the injection sites (see below and **Table 2**).

### Neuronal Tracing and Tissue Fixation

Animals were anesthetized as described above and fixed in a stereotaxic frame. After a medial incision of the scalp, a small hole was drilled into the skull with a dental drill. After cutting the dura mater, a glass capillary (tip diameter < 1 μm) was inserted and the tracer was slowly pressure-injected. The injection methods were optimized in our laboratory and used in a number of anterograde and retrograde tracing studies [e.g., (37–40)]. This included backfilling of the capillary, the careful cleaning of the tip, and pulling back a small amount of fluid before insertion. The injection coordinates were taken from the rat brain atlas (35), and are given in **Table 2**.

The *retrograde* tracer Fluoro-Gold [FG, 150 nl, 5 % in distilled water, Fluorochrome, Englewood, CO, USA, (41)] was injected into either the primary somatosensory (S1) cortex or the anterior cingulate cortex.

The *anterograde* tracer Phaseolus vulgaris-leucoagglutinin (Pha-L, 200 nl, 2.5% diluted in 0.1 M phosphate-buffered physiological saline (PBS), Vector, Burlingame, CA, USA) was injected into either the parafascicular nucleus (PaF), or the posteromedial/posterolateral ventral nuclei of the thalamus (VPM/VPL). The anterograde tracer Fluoro-Ruby (FR, 100 nl, 10% in PBS; Fluorochrome) was injected into the dorsomedial part of the laterodorsal thalamic nucleus (LDDM).

After 5–7 days, the animals were killed by anesthesia overdose and immediately perfused transcardially with PBS containing 15,000 IU heparin/l followed by 300 ml of ice-cold PLP solution (4% paraformaldehyde, 1.37% L-lysine, 0.21% sodium-periodate in PBS); according to McLean and Nakane (42) at a constant rate of 25 ml/min. The brains were removed and postfixed overnight in PLP, then cryoprotected in phosphate-buffered 30 % sucrose. They were marked on one side and sectioned serially at 30 mm thickness on a freezing microtome in the frontal plane, and collected in PBS into four parallel sets of sections. One set was directly mounted in order of appearance on gelatinized glass slides, dried, cleared in xylene, and coverslipped with Merckoglas (Merck, Darmstadt, Germany) for injection site verification. It was found that the tracer destination site was hit in at least three of five animals of each of the five injection groups. Sections stemming from these rats were analyzed further.

To visualize tracing, sections of one set were incubated free-floating with primary antibodies directed against Pha-L (1:1000 in PBS, raised in rabbit; Vector), while the autofluorescent substances Fluoro-Ruby and Fluoro-Gold do not require detection by antibody. Selected sections containing injection or target sites were counterstained with hematoxylin-eosin.

At least 10 sections per set from each of three rats of either injection group were used for immunohistochemistry. Further brain sections were used for antigen distribution and antibody specificity tests.

### Immunohistochemistry

Sections exhibiting labeling of terminal fields in cortical regions upon application of an anterograde tracer into the thalamic nuclei were incubated with an antibody directed against the μ-opioidergic receptor (guinea pig-anti-MOR-1, 1:2500, Chemicon, Temecula, CA, USA).

Sections from thalamic regions which exhibited labeling of neuronal perikarya after retrograde tracing from cortical regions were incubated with primary antibodies directed against either tyrosine-hydroxylase (mouse-anti-TH, 1:1000, Chemicon), serotonin (rat anti-SER, 1:200, Chemicon), neuronal nitric oxide-synthase (rabbit anti-nNOS, 1:1000, Progen, Heidelberg, Germany), or substance P (rat anti-SP, 1:200, Abcam, Cambridgeshire, England). Sections were incubated in primary antibodies diluted in PBS to which 0.1% Triton X-100 and 3% normal donkey serum were added. Immunoreactions were visualized with Cy2- or Cy3-conjugated F(ab)_2_-fragment of IgG directed against the host species of the primary antibody (1:200–1:400 in PBS, Jackson ImmunoResearch, Newmarket, Suffolk, England). Immunohistochemical specificity studies, carried out by omitting primary or secondary antibodies or by absorbing the primary antibody with the immunogen, showed the absence of the immunofluorescent signal. The antibodies had been used earlier in our laboratory and were characterized previously. Further details are given in Reuss et al. (43).

All sections were analyzed using an Olympus BX51 research microscope equipped with epifluorescence unit, highly specific single and dual band filter sets allowing the single or simultaneous excitation and observation of dyes without overlapping-artifacts (Olympus fluorescence monochromatic and dichromatic mirror cubes, maximal excitation/maximal emission, Cy2: 489/506 nm, Cy3: 552/565 nm). Photomicrographs were taken with a digital color camera employing the Analysis Software (Soft Imaging System, Münster, Germany). The Adobe Photoshop and Powerpoint programs were used to arrange images, to adjust image contrast and brightness, and to add labels.

## Results

Design and time course of the present study are given in Figure 1. In the first part (Figure 1A), rats with either thalamic lesions or sham-lesions were subjected to five PET-scans in the course of 21 days. MRI (Figure 1A_1_) and histology (Figure 1A_2_) verified lesions. In the second part (Figure 1B), neuronal tracing was used to identify connections between thalamic and cerebro-cortical regions. Immunohistochemistry was used for the detection of selected neuroactive substances in these regions. Delineations, nomenclature and abbreviations of thalamic nuclei, subcortical and cortical regions were adopted from the stereotactic rat brain atlas by Paxinos and Watson (35).

### First Experimental Series: PET Study

The effects of GVS in the unlesioned animals closely resembled those found in our previous study on the vestibular activation pattern in the two cerebral hemispheres (30). We then compared the effects of GVS in lesioned animals with those in sham-lesioned animals by two-sample-*T*-test and found time-after-lesion-dependent activations of distinct regions in lesioned animals (see Figure S1 and Table S1).

### Group Analyses; Intact Vestibular Stimulation vs. Lesion Time Frames (Paired *T*-Tests)

**Figure 2:**
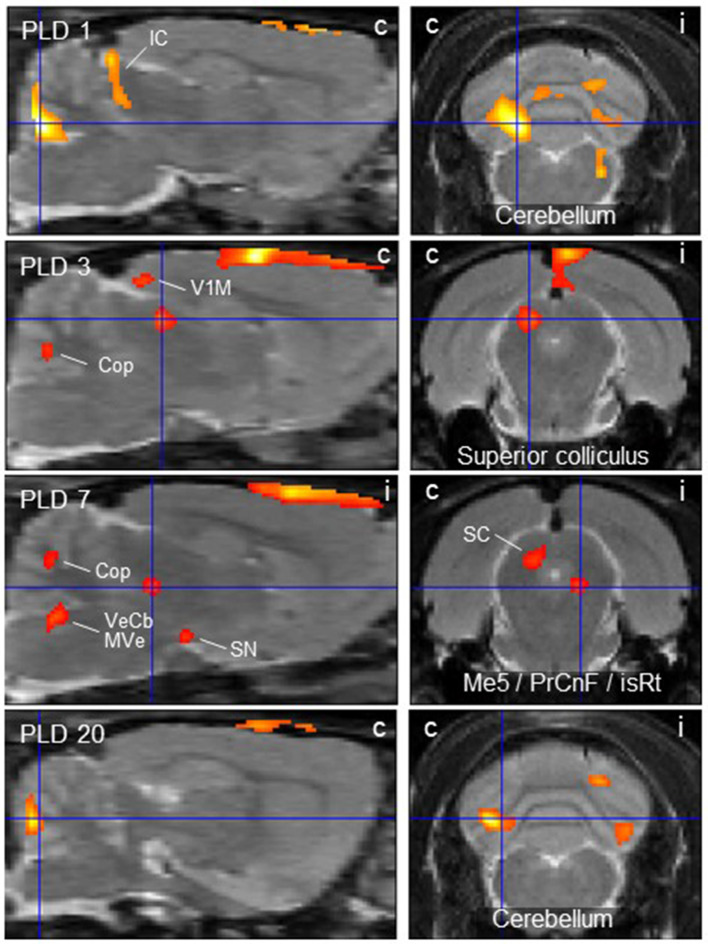
Thalamic lesion activates vestibular stimulation-induced glucose consumption in distinct brain regions. Clusters of higher metabolism upon galvanic vestibular stimulation after thalamic lesion compared to pre-lesion (paired *T*-test), at post-lesion days (PLD) 1–20. Shown are parasagittal (left) and coronal (right) sections. Orientation (i/c) = ipsi-/contralateral to lesion and GVS site. Cluster parameters are given in Table 1. Cop, copula pyramis; IC/SC, inferior/superior colliculus; isRt, isthmic reticular formation; Me5, mesencephalic trigeminal nucleus; MVe medial vestibular nucleus; PrCnF, precuneiform area; SN, substantia nigra; V1M primary visual area, monocular region; VeCb, vestibular cerebellar nucleus. The dorsalmost signal mainly outside brain parenchyma is an artifact related to lesion-surgery.

**Figure 3:**
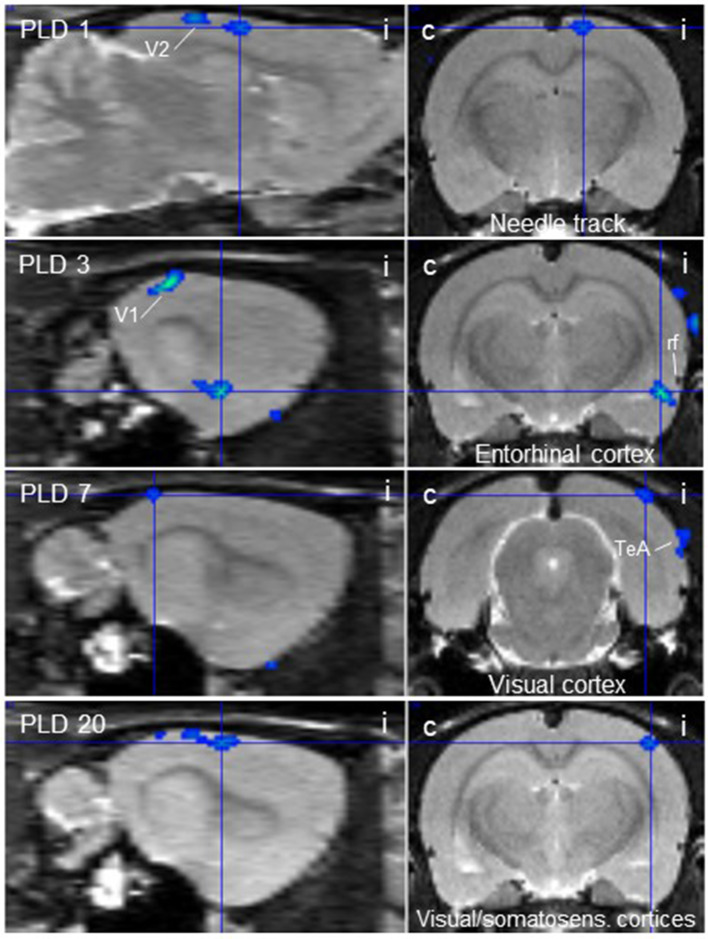
Thalamic lesion inhibits vestibular stimulation-induced glucose consumption in distinct brain regions. Clusters of decreased metabolism upon galvanic vestibular stimulation after thalamic lesion compared to pre-lesion (paired *T*-test), at post-lesion days (PLD) 1–20, as seen in parasagittal (left) and coronal (right) sections. i/c = ipsi-/contralateral to lesion and stimulation site. TeA, temporal association cortex; V1, primary visual cortex; V2, secondary visual cortex. Cluster parameters are given in Table 1.

Scans before lesion were compared with post-lesion scans at different post-lesion days (PLD) upon vestibular stimulation of all animals of the lesion group. Brain areas with augmented metabolism after lesions as well as areas with diminished metabolism were identified (Table 1; Figures 2, 3).

**Table 1:** Regions showing more (“activation,” positive Z-score) or less (“deactivation,” negative Z-score) rCGM augmentation upon galvanic vestibular stimulation at post-lesion days (PLD) 1–20, compared to pre-lesion levels (paired *T*-test).

**PLD**	**Brain region**	**Center of cluster coordinates (X/Y/Z)**	**Cluster size**	***Z*-score**	**Figures**
**Activations**					
1	Inferior colliculus, contra Cerebellum, contra	−2.6 / −9.8 / −2.6 −2.0 / −13.6 / −5.8	273 2019	+ 3.97 + 4.44	2
3	Superior colliculus, contra	−1.4 / −7.0 / −3.6	150	+ 3.96	2
7	Me5 / PrCnF / isRt, ipsi Superior colliculus, contra	1.2 / −7.4 / −5.4 −1.2 / −7.2 / −4.0	67 214	+ 3.85 + 3.71	2
20	Cerebellum, contra	−3.4 / −13.6 / −5.4	522	+ 4.74	2
**Deactivations**					
1	Needle track/lesioned area	1.8 / −2.4 / −0.6	123	– 3.67	3
3	Entorhinal cortex, ipsi	6.2 / −4.0 / −8.2	162	– 4.47	3
7	Visual cortex, ipsi	4.8 / −7.2 / −1.0	51	– 3.48	3
20	Visual cortex, ipsi Somatosensory cortex, ipsi	5.2 / −3.4 / −1.6	200	– 3.73	3
**Activability loss at PLD 20**					
1–20	Ventral medial geniculate body / Peripeduncular nucleus, contra	– 3.2/– 5.8/– 6.6	138	+ 4.7	4
1–20	Somatosensory cortex, contra	2.8/– 0.6/– 2.0	1635	+ 5.17	4

**Figure 4:**
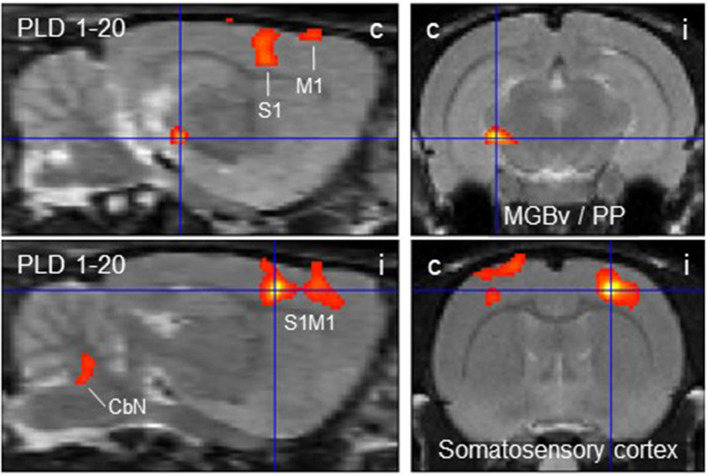
Distinct brain regions that were more activated upon galvanic vestibular stimulation at post-lesion day 1 (PLD 1) than at PLD 20. Clusters of higher metabolism at PLD 1 compared to PLD 1 (paired *T*-test) were found contralaterally in the ventral part of the medial geniculate nucleus/peripeduncular nucleus and bilaterally in the somatosensory cortex, as demonstrated in parasagittal (left) and coronal (right) sections. Deactivations were not observed under these conditions. i/c = ipsi-/contralateral to lesion and stimulation site. CbN, cerebellar nuclei; M1, primary motor cortex; PP, peripeduncular nucleus; S1, primary somatosensory cortex; MGBv, ventral aspect of medial geniculate body.

*Cluster size > 50 vx. Coordinates relate to Paxinos and Watson (35). X, lateral to midline; Y, anterior or posterior to Bregma; Z, below surface, in mm. Listed are clusters with high Z-scores, as depicted in Figures 2–4. Z-score is the position of a raw score in terms of its distance from the mean, measured in standard deviation units. Ipsi and contra refers to laterality with respect to lesion and stimulation. Me5, mesencephalic trigeminal nucleus; PrCnF, precuneiform area; isRt isthmic reticular formation. See results for more details*.

At PLD 1, activity was detected in the cerebellum bilaterally, with a contralateral dominance in the paramedian lobe and in the ipsilateral crus 2. The major cluster, located contralateral, also includes the copula of the pyramis, and parts of the vermis (lobules 8–10) (Figure 2). The area containing the electrode tract and the lesion, as well as the secondary visual area (V2), exhibited decreased activity (Figure 3).

At PLD 3, activity was augmented contralateral to the lesion in the superior colliculus, in the copula pyramis mainly contralateral and the contralateral retrosplenial primary visual cortex (V1, monocular area). It was diminished ipsilateral in the V1 area and in a cluster centered in the entorhinal cortex and partly covering aspects of ventral perirhinal and dorsal piriform cortices, as well as dorsal endopiriform and lateral amygdaloid nuclei.

At PLD 7, increased activation upon GVS was detected in the superior colliculus contralateral, in the bilateral cerebellar copula, and an activation cluster containing the closely located vestibular cerebellar nucleus and medial vestibular nucleus of the brainstem. Ipsilateral, the substantia nigra and a smaller mesencephalic cluster apparently including trigeminal nucleus, precuneiform area and isthmic reticular formation (Figure 2). Less activation was observed in the ipsilateral visual cortex and temporal association cortex (Figure 3).

**Figure 5:**
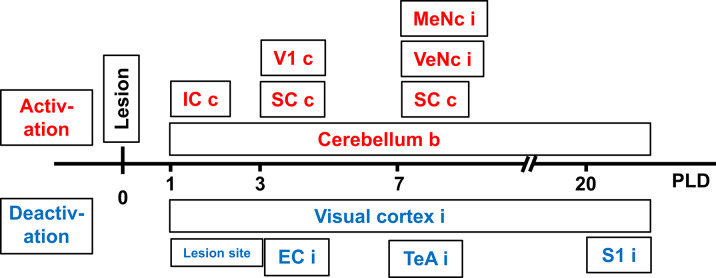
Time-course of augmented or reduced activation of metabolic activity in rat brain upon galvanic vestibular stimulation (GVS), at postlesion (PLD) days 1–20 compared to prelesion. EC, entorhinal cortex; IC, inferior colliculus; MeNc mesencephalic nuclei; SC superior colliculus; S1 primary somatosensory cortex; VTeA, temporal association cortex; V1, primary visual cortex; VeNc, vestibular nuclei; b, bilateral, c, contralateral, i, ipsilateral to lesion and stimulation.

At PLD 20, a similar activation cluster as at PLD 1 appeared in the cerebellum, again with a clear contralateral predominance and smaller clusters ipsilateral. Deactivations upon GVS were present in ipsilateral somatosensory and visual cortical area. These results are summarized in Figure 5.

In the parasagittal images of PLD 1-20 (Figure 2), an “activation band” was observed as an artifact dorsal to the cortical surface, mainly outside brain issue, that most probably is due to disturbed blood flow following lesion surgery (see discussion).

The subtraction of activation patterns of PLD 20 from PLD 1 demonstrated higher activity 1 day after lesion in the contralateral ventral aspect of the medial geniculate body and peripeduncular nucleus, as well as primary somatosensory and primary motor cortical area bilaterally, with an ipsilateral predominance (Figure 4). In addition, cerebellar nuclei showed a small activation cluster. Clusters of lower activity upon GVS were not apparent.

### Lesion Verification

Upon magnetic resonance imaging (MRI), pixels representing the needle track were visualized by a superposition of the images to a T1-weighted data set. The analysis of the lesion setting revealed the stereotactically correct insertion of the electrodes (Figure 1A_1_). The histological control of lesions, conducted after the final PET-scan (Figure 1A_2_) to confirm MRI-findings, showed that the main part of the LDDM and the dorsal and medially located aspects of the LDVL were damaged.

### Second Experimental Series: Neuroanatomical Study

#### Neuronal Tracing

**Figure 6:**
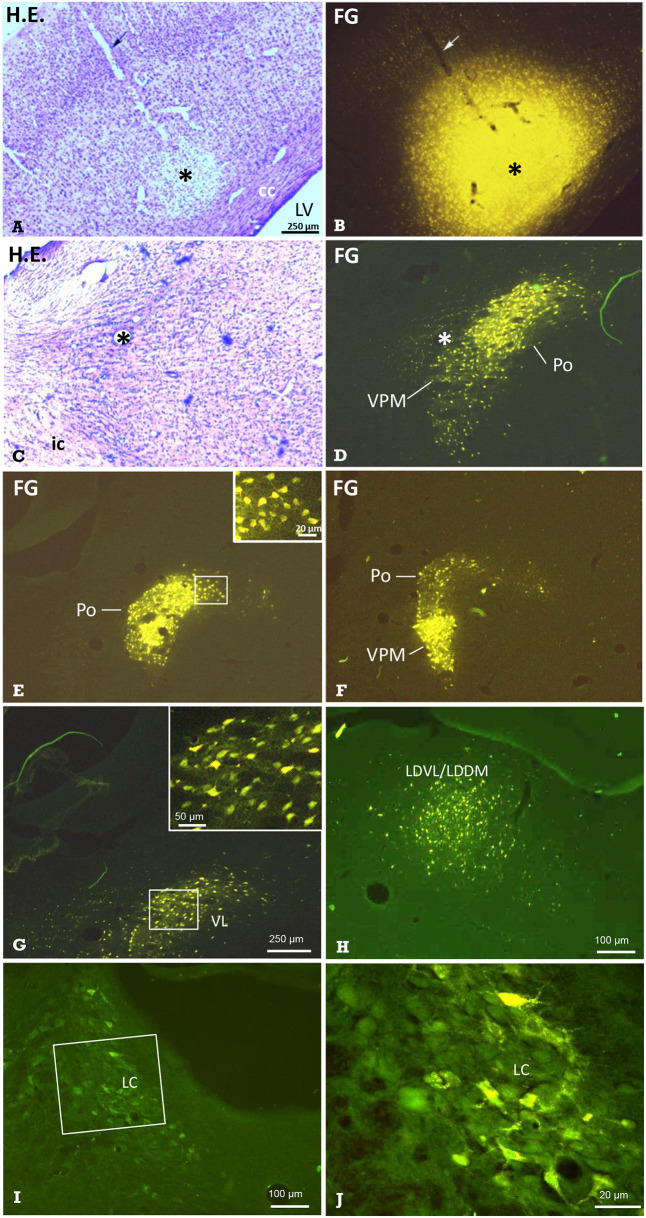
Identification of thalamic nuclei projecting to the cerebral cortex by retrograde labeling. Frontal sections showing the site of FG-injection and retrogradely labeled neurons in thalamic nuclei. **(A)** Hematoxylin-eosin-(H.E.) staining and **(B)** FG fluorescence imaging of the same section demonstrating a typical injection into S1. Arrows point to the pipette track. In the same animal, retrogradely labeled neuronal somata were observed in the **(C,D)** ventral posteromedial thalamus and posterior thalamic nucleus (VPM/Po). E Labeled neuronal somata in Po, and F in Po and VPM of the same animal, as well as in the ventrolateral thalamic nucleus (VL in **G**), ventrolateral and dorsomedial parts of the laterodorsal thalamic nucleus (LDVL/LDDM upon injection into ACC, in **H**), and in the locus coeruleus (**I**; boxed area is shown in higher magnification in **J**). Dorsal is up, lateral is left **(A–J)**. All labeled neurons were seen ipsilateral to the injection site. Marker bar in A applies to **(A–F)**. The insert in E enlarges labeled neuronal somata from the medial aspect of the Po. Asterisks in **(A–D)** mark the same structures. Approximate levels with regard to Bregma are: **(A,B)** −2.5 mm [according to Figure 54 of the rat stereotactic atlas (35)], **(C,D)** −3 mm (Figure 58), **(E)** −2.5 mm (Figure 54) and **(F)** −4.4 (Figure 70), **(G)** −2 mm (Figure 50), **(H)** −2.7 mm (Figure 56), **(I,J)** −9.7 mm (Figure 114). See also Table 2 for details. cc, corpus callosum; ic, internal capsule; LV, lateral ventricle.

**Figure 7:**
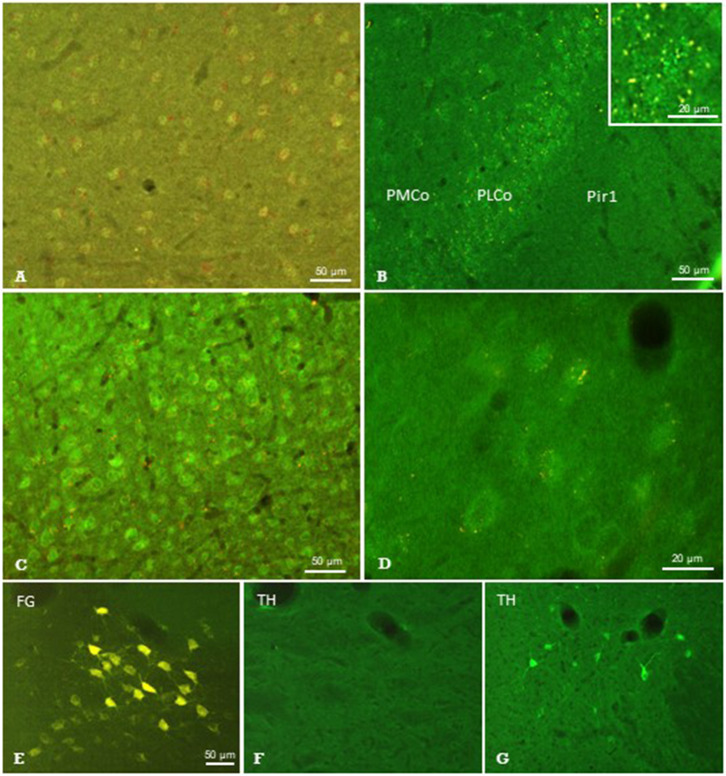
Results of neuronal tracing combined with immunohistochemistry. Injection of Pha-L into the thalamic parafascicular nucleus (PaF) resulted in anterogradely labeled axonal terminals (yellow-red) at neuronal somata exhibiting faint μ-opioidergic receptor-immunoreaction (green fluorescence) in the ipsilateral entorhinal cortex **(A)**, amygdala **(B)** and primary somatosensory cortex **(C,D)**. Retrogradely labeled PaF neurons upon FG injection into S1 **(E)**. These neurons did not show immunolabeling for tyrosine-hydroxylase (TH; **F**), which was, however, present in the hypothalamus in the same section **(G)**. FG, Fluoro-gold; Pir1, piriform cortex layer 1; PLCo/PMCo, posterolateral/posteromedial cortical amygdaloid nucleus. Brain surface is up.

Sections from 15 rats exhibiting successful injection of the neuronal tracers were analyzed. The results (consistent between all investigated rats) are summarized in Table 2, and examples are given in Figures 6, 7. We did not observe any spilling of the tracers into ventricular cerebrospinal fluid.

**Table 2:** Labeled brain regions upon neuronal tracing.

**Tracing type**	**Tracer substance**	**Injection site (center coordinates, X/Y/Z)**	**Tracer target site**	**IHC**	**Figures**
Retrograde	FG	S1 (4.8/−2.5/3)	VPL, VPM, VL, Po, PaF, In, LC, AuC2, S1 contra	TH-neg. somata	7
	FG	ACC (0.5/−1.5/1.7)	In, LDDM, LDVL		6
Anterograde	Pha-L	PaF (1.4/−4/5.7)	In, Hip (CA1), Amyg, EC, ACC S1	MOR-pos. somata	7, Suppl.2
	Pha-L	VPM/VPL (3/−3/5.8)	S1, ACC In, AuC		
	FR	LDDM (2/−2.5/4.5)	ACC		

*For abbreviations, see list. Injection site coordinates relate to Paxinos and Watson (35). X, lateral to midline; Y, anterior or posterior to Bregma; Z, below surface, in mm*.

The *retrograde* neuronal tracer Fluoro-Gold (FG) was used to label cell bodies of neurons projecting to distinct cortical regions such as the primary somatosensory cortex (S1) or anterior cingulate cortex (ACC) (Figure 1B_1_). After injection of FG into S1 (Figures 6A,B), distinct labeling of neuronal perikarya was found. They were located in groups with clear-cut borders. The comparison with Nissl-sections stained subsequently to fluorescence shooting and/or with those from the stereotactic rat atlas (35), revealed that they were located in the ipsilateral ventral posteromedial and posterolateral (VPM / VPL; Figures 6C,D,F), posterior (Po; Figures 6E,F), and ventrolateral (VL; Figure 6G) thalamic nuclei. While labeled neurons were densely packed in the VPM, they were more scattered in the VPL. We also found labeled neuronal somata in the ipsilateral granular, agranular, and disgranular insular cortical regions, in the locus coeruleus (LC; Figures 6I,J), and in the secondary auditory cortex. Some labeled somata were also found in the contralateral S1-region.

The injection of FG into the ACC resulted in retrogradely labeled neuronal perikarya in ipsilateral granular/disgranular insular cortex and in the thalamic LDDM/LDVL (Figure 6H).

The *anterograde* neuronal tracers Phaseolus vulgaris-Leucoagglutinin (Pha-L) and Fluoro-Ruby (FR) were used to label axonal projections to the cerebral cortex originating in thalamic nuclei (Figure 1B_2_). Prior experiments showed that thalamo-cortical tracing results were similar regardless of the anterograde tracer substance used. Injections were directed to either the PaF, VPM/VPL, or LDDM. Results of PaF-injections are given in Table 2 and depicted in Figures 7A–D and Figure S2. Fiber and/or terminal labeling was found in ipsilateral cerebro-cortical regions such as entorhinal cortex (Figure 7A), amygdala (Figure 7B), and primary somatosensory cortex (S1) (Figures 7C,D), as well as in ACC, hippocampal CA2 region and posterior agranular insular cortex (Figures 7A–D, Figure S2). The injection of FR into the LDDM caused labeling of fibers and terminal fields in the ACC. The injection of Pha-L into the posteromedial and posterolateral ventral thalamic nuclei (VPM/VPL) resulted in labeling of terminal fields in the primary somatosensory cortex, ACC, insular and auditory cortex.

### Immunohistochemistry on Labeled Structures

Sections showing successful neuronal tracing of perikarya or terminal fields, respectively, were subjected to immunohistochemical incubations in order to identify putative neurotransmitter systems (Figure 1C). The immunofluorescent detection of the μ-opioid receptor (MOR) resulted in labeled neuronal perikarya in cerebrocortical regions such as the entorhinal cortex (Figure 7A), amygdala (Figure 7B), and primary somatosensory cortex (Figure 7C) in each of the animals investigated. In this region, we observed terminal boutons anterogradely traced upon Pha-L-injection into the PaF in close vicinity to and covering MOR-positive neuronal somata (Figures 7C,D).

From the antibodies used, those directed against serotonin, TH, nNOS, or SP did not identify any structures labeled by antero- or retrograde tracing. The presence of respective immunofluorescence in the same section or in adjacent regions, however, validated our methodical approach. An example is given in Figures 7E,F showing the absence of TH-immunofluorescence in PaF thalamic neuronal perikarya retrogradely labeled upon FG-injection into S1, while immunoreactive neurons were present in subcortical regions in the same brain section (Figure 7G).

## Discussion

This rat study is the first to use functional brain imaging for the investigation of vestibular processing in the acute and subacute course following unilateral experimental lesion of a central vestibular site in the dorsolateral thalamus. It aimed at the processes underlying the clinical phenomenon known as “vestibular compensation (VC)” that includes a behavioral recovery after acute unilateral peripheral or central lesions within the vestibular system in patients [for reviews, see (15, 44, 45)]. For that purpose, we conducted PET-scans of the rat brain after lesion of the laterodorsal thalamic subnuclei (LDDM, LDVL). We also sought to add further knowledge to the subcortical-cortical pathways known to process vestibular information by bidirectional neuronal tracing of thalamo-cortical connections upon injections into involved sites.

In the first experimental series, we scanned rat brains by FDG-PET at five time-points. After the first scan, animals were subjected to an electrolytic lesion of the thalamus to interrupt the relay station of the inner ear-vestibular cortex chain. The PET-scans were conducted employing the galvanic vestibular stimulation (GVS) paradigm as used in our previous study (30). In intact animals, it widely mimics the physiological situation of vestibular challenge in anesthetized animals (31).

Thalamic lesions have the advantage that they leave the peripheral vestibular organ intact. They avoid tympanic membrane and ossicle removal as well as lymphatic breakdown and render the auditory system operating, also minimizing respective artifacts. This paradigm serves as a model for human thalamic infarcts, which often cause acute vestibular symptoms and thus are a major object of clinical therapy [cf. (11, 46, 47)]. Since we previously found in rats that vestibular processing was primarily resident in the left hemisphere regardless of the side of GVS (30), thalamic lesions were conducted at the left side in the present study.

We observed altered metabolism upon GVS in distinct brain regions after the lesion. That these effects were not a by-product of surgery was revealed by the comparison of lesioned animals to the group of sham-lesioned rats, which showed clear-cut differences under otherwise identical surgical procedures (see Supplementary Material). It should be emphasized here that we did not aim at the effects of GVS on the activation pattern in both hemispheres as in our previous study (30) but rather sought to identify structures that were altered in metabolism, and the time-course of these changes, by the experimental thalamic lesion. This lesion site represents an important relay and integration area of vestibular projections en route to the cortical vestibular circuitry that was characterized by diffusion tensor imaging and connectivity MRI studies in healthy participants more recently (48, 49). We will discuss the different findings in their context in the following.

### Vestibular Stimulation Pre- vs. Post-Lesion (Paired *T*-Test)

The most important findings of our PET-scan analysis were based on paired *T*-tests comparing brain metabolic activity after the unilateral lesion with pre-lesion data in single animals, in each case upon GVS. The differences at a given PLD were then averaged over all animals of the group. As summarized in Table 1 and shown in Figure 2, we found major clusters of augmented metabolism, at different times after lesion, in the cerebellum and superior colliculus, either bilaterally or particularly contralateral to the lesion site. We also found activation after lesion in the visual cortex, inferior colliculus, and vestibular nuclei, although to minor degrees. These regions are part of the multisensory vestibular network [cf. (45, 50, 51)]. Diminished metabolism was found in ipsilateral entorhinal, visual, and somatosensory cortical areas. This is in line with the concept of a reciprocal inhibitory interaction between the sensory systems, i.e., activation of the vestibular system by GVS and downregulation of the visual and somatosensory systems (52), and was recently supported by imaging data on multiple central vestibular pathways that exert “large-scale modulatory effects of the vestibular system on sensory processing” (53).

The cerebellum showed activated metabolism during the period of study, with decreasing magnitude from PLD 1 to PLD 20. A major cluster was located contralateral, while smaller clusters were activated in the ipsilateral copula pyramis and lobules 8–10 of the vermis, thus covering regions known as part of the vestibulocerebellum [cf. (54)]. This brain structure is a major relay in the processing of vestibular input to control and fine-tune stabilization of the eyes, head, and body during motion. The vestibulocerebellum is a direct target of primary or secondary vestibular input in mammals including man [cf. (45, 55)], and it projects back to the brainstem vestibular nuclei and to the cerebellar fastigial nuclei, an output relay station, where units respond to GVS (56). There is also evidence that the cerebellum is important in compensation of vestibular deficits (57), which was supported, inter alia, by the finding that mice lacking cerebellar Purkinje (output) neurons recovered less efficiently from vestibular lesions than wild-type mice (58). The vestibulocerebellum, notably, was also among the structures exhibiting increased rCGM contralateral to labyrinthectomy in rats (22). Enhanced cerebellar activations were in parallel also found in patients with unilateral infarctions of the medulla affecting the VN (28) and of the midbrain (29). Augmented cerebellar metabolic activity in the present study may be attributed to increased processing effort due to incongruent vestibular inputs in both hemispheres, i.e., intact input via vestibular nerve and pathways in one hemisphere and interrupted input via the lesioned thalamic pathway in the other.

The second structure activated by GVS in lesioned animals, the superior colliculus, is strongly involved in the control of eye movements during vestibular processing. In the comparison lesion vs. sham-lesion (see Supplementary Material), it showed decreased metabolic activity at day 1 and, in the pre- vs. post-lesion analysis, at days 3 and 7. These results may indicate a compensatory shift to visual signaling due to the damage of vestibular signaling. Such a substitution by one sensory system in case of a lesion in another is known from acute as well as chronic vestibular disorders (27, 59) in several systems. This is further supported by our present finding that the ipsilateral visual V1/V2 regions were deactivated at PLD 1 to 20, while the contralateral (monocular) primary visual area was activated.

In addition, the ipsilateral entorhinal, somatosensory, and temporal association cortices exhibited diminished activation upon GVS in the post-lesion period which may be explained by reduced vestibular (and putatively other esp. somatosensory) input upon thalamic lesion and therefore reduced intersensory interaction within the hemisphere. Moreover, the thalamic damage not only affects vestibular, but also somatosensory and visual thalamo-cortical afferents directly. This reduced activation was not observed contralateral to the lesion where the pathway was not interrupted. It appeared that, in most regions, the activation by GVS was reduced at the side of lesion, while it was rather augmented contralateral. There were, however, a few ipsilateral regions activated by GVS, but these are integrated “upstream” to the lesion in the pathway under investigation. They include a relatively small mesencephalic cluster covering the trigeminal nucleus, precuneiform area and isthmic reticular formation, which provide functional features such as intrathalamic relay, midbrain locomotion center, and reticulocortical projections, respectively (60). Further activated were the ipsilateral substantia nigra and, notably, vestibular nuclei of cerebellum and brainstem.

With regard to the “activation band” dorsal to the cortical surface, mainly outside brain tissue, as visible in the parasagittal images of PLD 1-20 (Figure 2), it should be pointed out that this is most probably an artifact that is due to disturbed blood flow and respective regeneration processes following lesion surgery involving craniotomy and cutting of the dura mater. The “signal” was not seen in prelesion-PET presently and in our previous paper (30), which did not include surgery.

It is believed that these cortical sites belong to the network of cortical regions involved in vestibular processing. In rats, they include primary and secondary somatosensory, infralimbic and cingulate cortices (30, 61). In monkeys, important sites are area 7 as well as the parieto-insular vestibular cortex (PIVC) (7, 8, 62, 63). In humans, the posterior insular cortex, retroinsular region, parietal operculum, somatosensory Brodmann areas 2 and 3a, superior temporal gyrus, inferior-parietal gyrus, anterior cingulate cortex, and the hippocampus belong to this network (1, 9, 64, 65). Metaanalyses of human imaging studies support the view that the posterior insula, retroinsular region, and parietal operculum may represent the core region PIVC in monkeys (66, 67).

This above-mentioned network shows functional dichotomy as (1) vestibular projections diverge into a network of several cortical regions and (2) multisensory information converges in distinct cerebral areas such as the vestibular nuclei and thalamus. This implies also that respective brain regions such as vestibular cerebellum, thalamus, and cortex are not exclusively in charge for vestibular processing but are multisensory and handle also extravestibular inputs [cf. (45, 68)]. Based on previous and actual findings, we used neuroanatomical methods to add further knowledge on the connections of involved structures.

### Neuronal Tract Tracing

Since the S1 region was the most dominant structure upon GVS in our previous PET-study (30), we conducted retrograde neuronal tracing upon unilateral injection of FG into this region and thereby labeled neuronal perikarya in several thalamic nuclei. We then performed anterograde tracing upon unilateral Pha-L or FR injections into the thalamic VPL/VPM or PaF, and observed labeled terminals in several cortical regions.

Clear corticopetal projections to S1 originating in thalamic regions VPL, VPM, VL, Po, and LDDM were observed upon retrograde tracing presently. The scattered distribution of labeled neurons in the anterior VPM, VPL, and posterior Po and their rather dense appearance in anterior Po and posterior VPM suggest that the latter contribute stronger to S1-directed projections. Anterograde tracing confirmed the findings of retrograde tracing as labeled terminals were observed in S1 upon injection into VPM/VPL or PaF. Our findings support and extend thalamo-somatosensory cortical projections described previously upon lesion studies in monkeys (69) and upon anterograde neuronal tracing in rats (70). It is agreed to that the thalamus represents a subcortical site of multisensory integration. Many of its neurons (e.g., in laterodorsal nucleus, VPM, VPL, MGB) respond to vestibular, visual, proprioceptive, and somatosensory stimuli, and project to vestibular and non-vestibular cortical regions (71). Parenthetically, similar mechanisms of vestibular and extravestibular integration have been identified in the VN that provide projections to the thalamus. Ascending and descending in- and outputs of various cell branches account for the complexity of the vestibular network at this level, where the sensory input of location and motion depends on various other information such as gaze control, head-neck motion, and stabilization as well as fear, memory, and viscerosensory information (50, 72).

The characteristic of thalamic nuclei as vestibular relay station was also revealed previously by demonstration of connections from the VN to thalamic nuclei such as VPM (73, 74) and PaF (75–77), from the thalamus to somatosensory cortical regions, and by somatosensory backprojections to the thalamus (78) in rats. Several studies using functional brain imaging in man and rat showed the vestibular association of these connections (11, 30, 65). Functional connectivity MRI in healthy participants found two parts of the thalamus involved in vestibular processing, the paramedian subnuclei and the dorsolateral subnuclei (48, 49). These two regions processing vestibular information were also found to be causative in those patients with acute thalamic infarctions presenting with symptoms of an acute vestibular imbalance, i.e., either ipsilateral or contralateral tilts of the subjective visual vertical (79).

We also found projections to the S1-region originating from locus coeruleus as well as from secondary auditory and insular cortical regions. There is evidence that the LC is involved in vestibular processing since projections from VN to LC and vice versa have been shown previously (80, 81).

In addition to S1, the ACC is a target of parallel vestibular-related pathways. It receives projections from VPM/VPL and LDDM (our present retro- and anterograde tracings), and is activated by saccular or caloric stimulation (82, 83). The LDDM, part of the lateral thalamic complex, was previously identified by GVS as part of the vestibular network (30). Its neurons are directly innervated by medial vestibular nuclei neurons (75), to which the ACC in turn sends backprojections (84). Furthermore, recent tract tracing in mice revealed that the laterodorsal thalamus relays cerebellar projections to the hippocampus (85).

Finally, our injections into the PaF resulted in anterograde labeling of fibers and putative terminals in hippocampus, amygdala and entorhinal and insular cortices, which all showed altered metabolism upon GVS (30). Since entorhinal cortex and hippocampal CA1 harbor so-called place-cells and head-direction-cells coding a subject's location within the environment, it is likely that the vestibular input contributes to spatial information processing (86). The loss of this vestibular input resulted in hippocampal atrophy in patients with bilateral vestibular failure and deficits in spatial memory (87). In addition, effects upon experimental alterations of gravity revealed the involvement of the amygdala in vestibular processing (88, 89).

Special attention should be given to the insular cortex, the core region of the vestibular cortical network. Neurophysiological studies in animals (8, 90–92) and functional brain imaging in rat (30) and man (64, 65, 83, 93), imaging meta-analyses (66, 67) as well as functional connectivity studies in men (94) underline the importance of the insular-opercular area for vestibular processing, particularly in primates. A comparison of vestibular spatio-temporal tuning in macaque cortical areas PIVC, VIP, and MSTd showed a gradual transformation of temporal responses that suggest a hierarchy in cortical vestibular processing, with PIVC being most proximal to the vestibular periphery and MSTd being most distal (92). In line, neurons in the insular cortex were labeled upon retrograde tracer injections into the ACC in the present study demonstrating this tight connection. In humans, anterior and posterior insula, dorsolateral thalamus, ACC and cerebellum were activated by GVS, while visual and somatosensory cortices were simultaneously deactivated (65). Similar activations and deactivations were found in patients with vestibular neuritis predominantly in the contralateral hemisphere (95). Patients suffering from acute unilateral infarction of the posterolateral thalamic nuclei exhibited stronger activation of the inferior insula and reduced activation in the ipsilateral temporo-parietal cortex upon caloric stimulation, whereas the activation pattern was normal during caloric stimulation of the ear contralateral to the lesioned side (11). This affection of thalamo-cortical pathways within the lesioned hemisphere was now worked-up in more detail by retro-and anterograde tracer labeling in the current study.

However, the cortical aspects of metabolic alterations following thalamic lesion had not been in the focus of experimental studies yet. Recently, the time-course of rGCM upon surgical labyrinthectomy in rats was studied by PET-imaging (22). In the acute phase, VN, cerebellum, thalamus, hippocampus and amygdala were affected, while at post-lesion day 2 thalamus and cortex were inconspicuous, and glucose metabolism increased in vestibulocerebellum, amygdala, and hippocampus.

The present study combining brain PET-imaging upon thalamic lesions and retro- and anterograde neuronal tracing thus supports and extends earlier neuroanatomical and electrophysiological findings on the vestibular network.

### Immunohistochemistry Suggests Involvement of Opioids

Neuroactive substances found in the vestibular system belong to three chemical groups, i.e., amino acids, monoamines, and ACh, and neuropeptides. Their distribution was studied predominantly in the end organ, in Scarpa's ganglion and in VN, structures that are well-defined with respect to their functional identity. Studies of “central vestibular transmission” actually identified dopaminergic, serotonergic, nitrergic, and amino-acidergic transmitter systems of the VN [cf. (96–98)] while subcortical and cortical structures of the pathways upstream to the VN are less well-defined in terms of neurochemistry. However, many thalamic and cortical regions are multisensory and multimodal and thus may use various neuroactive substances for transmission.

Dopaminergic involvement in vestibular pathways was described previously. In the rat VN, D2-receptors are present, and there is evidence for noradrenergic input from locus coeruleus neurons [cf. (96)]. Notably, dopamine-receptor availability was reduced in temporo-parieto-occipital cortex, striatum and thalamus in patients with chronic bilateral vestibular failure, suggesting that lacking peripheral vestibular input led to receptor down-regulation in these regions (99). However, respective immunohistochemical data demonstrating the presence of these aminergic substances are missing. Our findings that thalamic neurons projecting to S1 did not exhibit immunoreactivity to TH, a marker enzyme of dopaminergic/noradrenergic synthesis, reveal that this part of the vestibular pathway is not dopaminergic. It should be noted that, similarly, serotonin, nNOS, and SP were not detected in structures identified by neuronal labeling in our study.

Several lines of evidence, on the other hand, suggest the presence of opioidergic mechanisms in vestibular pathways. The presence of enkephalins in vestibular efferent neurons (100) suggests that the end-organ is under opioidergic influence, probably via kappa-opioid receptors expressed in hair cells and μ-opioidergic receptors (MOR) in afferent synapses (101). There is further evidence for the involvement of enkephalins in the medial VN in vestibular processing and in compensational mechanisms (21), probably mediated by delta-opioid receptors on MVe neurons (102). Our finding that terminals of thalamic neurons were present at cerebrocortical neuronal cell bodies expressing MOR fits well to the modulation of opioid receptor availability by diprenorphine, an unspecific opioidergic antagonist in the human insular cortex during caloric stimulation in healthy volunteers (103).

It is obvious that the roles of these and other neuroactive substances in subcortical-cortical pathways warrant further studies on vestibular processing and compensation.

## Conclusions

Our previous rat data showing that GVS activates rCGM in distinct cortical and subcortical regions characterized those as part of a vestibular network. Our present data of a rat model with unilateral thalamic lesions demonstrate that GVS resulted in augmented activation of contralateral cerebellum and distinct subcortical structures such as superior colliculus as well as diminished activation of ipsilateral sensory cortical regions, showing a distinct pattern measured during 20 days post lesion. These rat data resemble the reduced activation of the ipsilateral cortex during caloric stimulation in patients with acute unilateral thalamic infarction (11). The changes in rCGM observed after lesion may be interpreted as brain plasticity mechanisms associated with vestibular compensation and substitution. Axonal tracings confirmed connections between subcortical and cortical sites identified in the PET-studies. These tracings combined with immunofluorescence showed that thalamic projections to S1 and ACC meet neurons expressing opioid receptors, but apparently do not involve neurotransmission based on serotonin, substance P, dopamine/noradrenaline, or nitric oxide.

## Data Availability Statement

The datasets generated for this study are available on request to the corresponding author.

## Ethics Statement

All applicable international, national, and/or institutional guidelines for the care and use of animals were followed. This article does not contain any studies with human participants performed by any of the authors.

## Consent for Publication

Consent to submit has been received explicitly from all co-authors.

## Author Contributions

SR: study concept and design, study supervision, analysis and interpretation of data, draft manuscript for intellectual content, and critical revision of manuscript for intellectual content. ES: acquisition of data, analysis and interpretation of data, and critical revision of manuscript for intellectual content. US, NB, NS, and AK: acquisition and analysis of data. H-GB: acquisition, analysis and interpretation of data, and critical revision of manuscript for intellectual content. MD: study concept and design, and critical revision of manuscript for intellectual content. MS: study concept and design.

## Conflict of Interest

The authors declare that the research was conducted in the absence of any commercial or financial relationships that could be construed as a potential conflict of interest.

## Abbreviations

^18^F-FDG: ^18^F-Fluorodesoxyglucose
ACC: anterior cingulate cortex
ACh: acetylcholine
Amyg: amygdala
AuC1/AuC2: primary/secondary auditory cortex
PBS: Phosphate-buffered saline
CA: cornu ammonis
CbN: cerebellar nuclei
Cop: copula pyramis
CPu: caudate putamen (striatum)
EC: entorhinal cortex
FOV: central field of view
FG: Fluoro-Gold
FR: Fluoro-Ruby
GABA: gamma-aminobutyric acid
GVS: galvanic vestibular stimulation
Hip: hippocampus
IC: inferior colliculus
IHC: immunohistochemistry
In: insular cortex
isRt: isthmic reticular formation
LC: locus coeruleus
LDDM: dorsomedial part of laterodorsal thalamic nucleus
LDVL: ventrolateral part of laterodorsal thalamic nucleus
M1: primary motor cortex
Me5: mesencephalic trigeminal nucleus
MGB: medial geniculate body
MGBv: ventral aspect of medial geniculate body
MOR: μ-opioid receptor
MRI: magnetic resonance imaging
MVe: medial vestibular nucleus
PaF: parafascicular nucleus of the thalamus
PET: positron emission tomography
Pha-L: Phaseolus vulgaris-leucoagglutinin
PIVC: parieto-insular vestibular cortex
PLD: post-lesion day
Po: posterior nucleus of the thalamus
PP: peripeduncular nucleus
PrCnF: precuneiform area
rCGM: regional cerebral glucose metabolism
S1/S2: primary/secondary somatosensory cortex
SC: superior colliculus
SL: Sham-lesion
SN: substantia nigra
TeA: temporal association cortex
TH: tyrosine hydroxylase
V1M primary visual area: monocular region
V1: primary visual cortex
V2: secondary visual cortex
VC: vestibular compensation
VeCb: vestibular cerebellar nucleus
VL: ventrolateral nucleus of the thalamus
VN: vestibular nuclei
VPL: ventral posterolateral nucleus of the thalamus
VPM: ventral posteromedial nucleus of the thalamus.
