# Supplementary material for: Modeling Vestibular Compensation: Neural Plasticity Upon Thalamic Lesion
Source: Front Neurol. 2020 May 22;11:441. doi: 10.3389/fneur.2020.00441 (PMC7256190; doi:10.3389/fneur.2020.00441)
Supplement: Supplementary file 1 [file Table_1.DOCX]

| **PLD** | **Brain region** | **Coordinates (X/Y/Z)** | **Cluster size** | **Z-score** | **Suppl. figure** |
| --- | --- | --- | --- | --- | --- |
| **1** | Postsubiculum, ipsi  Superior colliculus, ipsi (lateral aspects of deep layers) | 3.2/-7.2/-2.4  2.2/-6.6/-5.2 | 109  69 | **+** 4.19  **-** 3.91 | 1 |
| **3** | Medial geniculate nucl., contra (medial and ventral aspects) | -3.4/-5.2/-6.4 | 111 | **-** 3.81 | 1 |
| **7** | Entorhinal cortex (dorsolat.), ipsi Perirhinal cortex  Dentate gyrus, ventral, contra | 6.4/-5.8/-7.4 | 72 | **-** 3.72 | 1 |
| **20** | Zona incerta, contra | -2.4/-4.4/-7.8 | 201 | **+** 3.57 | 1 |

**Supplementary table 1.** Activated (positive Z-score) and deactivated (negative Z-score) brain region upon galvanic vestibular stimulation at post-lesion days (PLD) 1-20. (Lesion group compared to sham-lesion group; two-sample T-test). Coordinates relate to Paxinos and Watson (2014). X = left/right to midline, Y = anterior or posterior to bregma, Z = below surface. Note that positive Z-scores represent activations, negative Z-scores represent deactivations.
