# Supplementary material for: Modeling Vestibular Compensation: Neural Plasticity Upon Thalamic Lesion
Source: Front Neurol. 2020 May 22;11:441. doi: 10.3389/fneur.2020.00441 (PMC7256190; doi:10.3389/fneur.2020.00441)
Supplement: Supplementary file 2 [file Image_1.pdf]

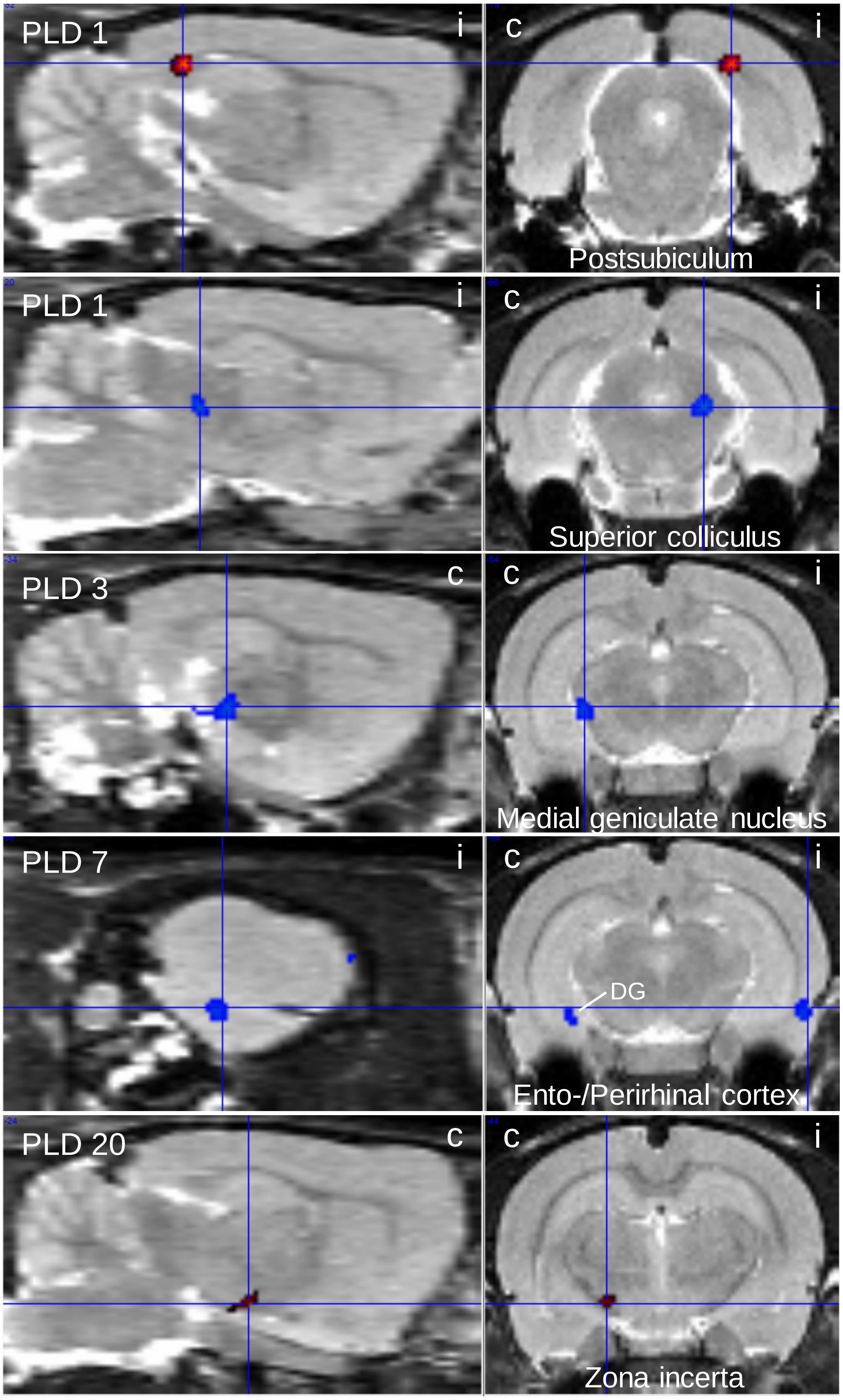

Suppl. Fig. 1. Thalamic lesion modifies vestibular stimulation-induced glucose consumption in distinct brain regions. Clusters of higher (red) or lower (blue) metabolism upon galvanic vestibular stimulation in the thalamus-lesion group compared to sham-lesion group (two-sample T-tests), at post-lesion days (PLD) 1 to 20. i/c, ipsi-/contralateral to lesion site. DG, dentate gyrus (ventral aspects)
