# Supplementary material for: Modeling Vestibular Compensation: Neural Plasticity Upon Thalamic Lesion
Source: Front Neurol. 2020 May 22;11:441. doi: 10.3389/fneur.2020.00441 (PMC7256190; doi:10.3389/fneur.2020.00441)
Supplement: Supplementary file 3 [file Image_2.pdf]

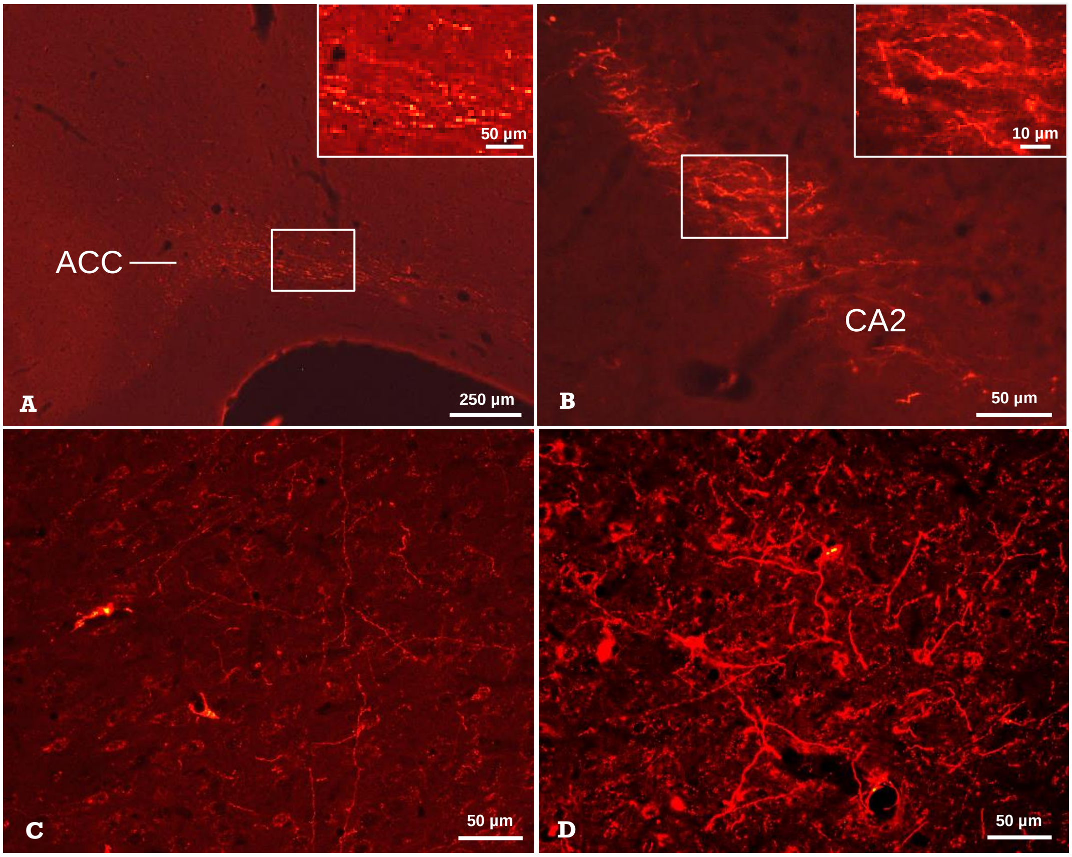

Suppl. Fig. 2. Anterograde neuronal tracing upon thalamic injection results in labeling of fibers and putative terminals in cortical regions. Upon Pha-L injection into the PaF, anterogradely labeled fibers and terminal fields in the anterior cingulate cortex (ACC) (**A**) and fibers in the hippocampal region CA2 (**B**) of the same animal. Inserts are magnifications of the boxed areas. Labeled fibers and putative terminals in the insular cortex (**C**) and the primary somatosensory cortex (**D**). Dorsal is up, lateral is left (**A-D**). All anterograde labeling was found ipsilateral to the injection site. Approximate levels are: **A-C** Bregma -2 mm, according to fig. 50 of the rat stereotactic atlas (Paxinos and Watson, 2014); **D** Bregma -2.7 mm (fig. 56).
